# *Colpodella* sp. (Phylum Apicomplexa) Identified in Horses Shed Light on Its Potential Transmission and Zoonotic Pathogenicity

**DOI:** 10.3389/fmicb.2022.857752

**Published:** 2022-04-18

**Authors:** Ming Xu, Yang Hu, Hongyu Qiu, Jingyuan Wang, Jiafu Jiang

**Affiliations:** ^1^School of Basic Medical, Inner Mongolia Medical University, Hohhot, China; ^2^State Key Laboratory of Pathogen and Biosecurity, Beijing Institute of Microbiology and Epidemiology, Beijing, China; ^3^College of Animal Science and Veterinary Medicine, Heilongjiang Bayi Agricultural University, Daqing, China

**Keywords:** *Colpodella* sp., horse, tick-borne pathogens, China, piroplasmosis

## Abstract

*Colpodella* species, which mainly feed on protists and algae, are free-living close relatives of apicomplexans. Recent reports have identified *Colpodella* sp. infections in an immunocompromised individual and a suspected case of tick-transmitted infection resulting in neurological symptoms. Our molecular examination of piroplasmosis-infected horses in China identified nearly whole 18S rRNA gene sequences that are closely related to *Colpodella* sp. ATCC 50594 isolated from brown woodland soil at Gambrill State Park, located in Frederick, MD, shedding light on an underreported emerging zoonotic pathogen.

## Introduction

*Colpodella* species are a group of small predatory flagellates that have close relationship with apicomplexans (Kuvardina et al., 2002). Most are free-living and feed on algae or protozoa (Leander and Keeling, 2003; Yadavalli and Sam-Yellowe, 2017), which are rarely reported in vertebrates and arthropod vectors. In 2012, the first human case of *Colpodella* sp. infection was reported with multiple ring form parasitic in erythrocytes and confirmed using molecular analysis (Yuan et al., 2012). It is also noted that the *Colpodella* sp. was detected in *Rhipicephalus microplus* infesting cattle in Mozambique, Africa (Matsimbe et al., 2017). In 2018, *Colpodella* spp. were further detected in ticks and confirmed in a human patient presenting with neurological symptoms (Jiang et al., 2018). Four sequences of *Colpodella tetrahymenae* and *Colpodella* sp. (GenBank accession nos. MH012044–MH012047) were also reported to be obtained from ticks (*Dermacentor everestianus* and *D. nuttalli*) collected in Qinghai Province, China. Five partial sequences (∼580 bp 18S rRNA) of *Colpodella* sp. (accession nos. MN640805–MN640809) were also reported to originate from Amur tiger (*Panthera tigris altaica*) in GenBank. The relatively wide distribution of the genus *Colpodella* and the symptoms associated with infection should be considered a public health concern. Recently, during routine detection of equine piroplasmosis for horses, we accidentally found *Colpodella* sp. in horse blood. Here, we provided evidence of this potential pathogenic agent detected in horses and discussed the potential transmission to draw attention to the emergence of *Colpodella* as a potentially underreported parasitic disease in both humans and animals.

## Materials and Methods

### Sample Collection

Our original study design aimed to identify equine piroplasmosis in horse samples collected in close proximity to Ordos City, Inner Mongolia, located in northern China (longitude 106°42′40″–111°27′20″, latitude 37°35′24″–40°51′40″). Whole blood was collected using EDTA anticoagulant tubes, with collection location, time, and sample number recorded, prior to storage at –20^°^C.

### DNA Extraction and PCR Amplification

DNA was extracted from whole blood samples using Blood DNA Extraction Kits (Tiangen Biotechnique Inc., Beijing, China) following the manufacturer’s instructions. Obtained DNA were stored in a –20^°^C refrigerator while awaiting further testing.

A universal primer PIRO 0F (5′-GCC AGT CAT ATG CTT GTG TTA-3′) and PIRO 6R (5′-CTC CTT CCT YTA AGT GAT AAG GTT CAC-3′) (Kawabuchi et al., 2005) targeting 1,600 bp segment sequences of 18S rRNA gene of *Babesia* spp. and *Theileria* spp. were used to test for piroplasma infection. The PCR mixture consisted of 10.5 μl ddH_2_O, 12.5 μl 2 × Ex Taq Plus Master Mix, 0.5 μl of each primer, and 1 μl template DNA. An initial denaturation step at 95°C for 5 min was followed by 35 cycles of denaturation at 95°C for 1 min, annealing at 55°C for 1 min, and extension at 72°C for 1 min. A final extension was done at 72°C for 7 min, followed by a hold step at 4°C. PCR products were then detected by 1% agarose gel electrophoresis. To minimize risks of contamination, template isolation, PCR setup, and agarose gel electrophoresis occurred in separate rooms, in addition to including a negative control in each amplification.

### Sequence Determination

Positive amplicons were sent to Comate Biosciences Co., Ltd. (Changchun, China) for sequencing on a 3730 DNA Sequencer (Applied Biosystems, Foster City, CA, United States) from both directions. The bidirectional determination of the sequences were used for sequence splicing with SeqMan software. The clear sequences obtained were then compared with previously published sequences deposited in GenBank using the BLAST program from the National Center for Biotechnology Information website. Nucleotide sequences reported in this study were then deposited into GenBank with accession numbers.

### Alignments and Phylogenetic Analysis

The alignments of novel sequences, together with reference sequences from GenBank, were constructed with ClustalW using MEGA software (6.0). Phylogenetic trees were reconstructed using neighbor-joining, maximum likelihood, and maximum parsimony methods, respectively. Three analysis methods were performed using MEGA (6.0), with best fit selected according to the topology of the phylogenetic trees. Phylograms were drawn using the Fig Tree (v. 1.4.2).

### Statistical Analysis

Statistical analysis was performed by SPSS 22.0 software, and tick-borne pathogen risk factors were analyzed by χ^2^ test or Fisher’s exact test. Bilateral *p* < 0.05 means the difference is statistically significant.

## Results and Discussion

### Overall Infection Rates

**FIGURE 1:**
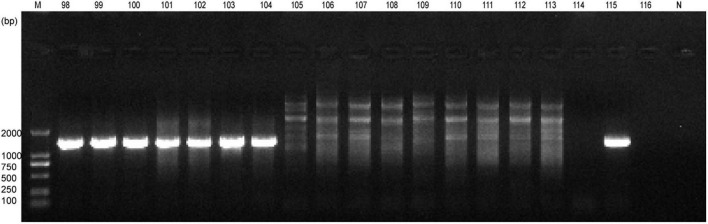
Results of electrophoresis of the amplicons for part samples by PCR with common primers. Lanes 98–116: the sample no. horse blood. N, negative control.

Whole blood samples were collected from a total of 400 horses and screened for piroplasma infection. A total of 136 (34.0%) samples were positive using common primers targeting 18S rRNA for piroplasms (Kawabuchi et al., 2005), which showed ∼1,600-bp amplicons under electrophoretic diagram (Figure 1). Entire sequences were obtained from positive amplicons. After conducting BLAST search, 132 (33%) horses were confirmed infected with *Theileria equi*, two were infected with *Babesia caballi*, and another two (0.5%), numbered NM103 and NM115, were infected with *Colpodella* sp. (Table 1). The infection rates of these three pathogens are significantly different in horses (*p* < 0.001, χ^2^ = 280.296). Analyses suggested that the phylogenetic position of *Colpodella* sequences fell within the sister group of the apicomplexan clade (Kuvardina et al., 2002), including *Babesia* spp., *Theileria* spp., *Toxoplasma* gondii, *Cryptosporidium* spp., and *Plasmodium* spp. Thus, it was not surprising to inadvertently amplify *Colpodella* with these common primers, similar to what was observed in a previous study (Jiang et al., 2018).

**TABLE 1:** Infection of three protozoa among horses collected in Inner Mongolia.

Sample site	Positive/tested (%)				
	*Colpodella* sp.	*Babesia caballi*	*Theileria equi*	Total	*P*-value
Ordos, Inner Mongolia	2/400 (0.5)	132/400 (33.0)	2/400 (0.5)	136/400 (34.0)	<0.0001*

*p < 0.05, the difference was statistically significant.

### Phylogenetic Analysis

**FIGURE 2:**
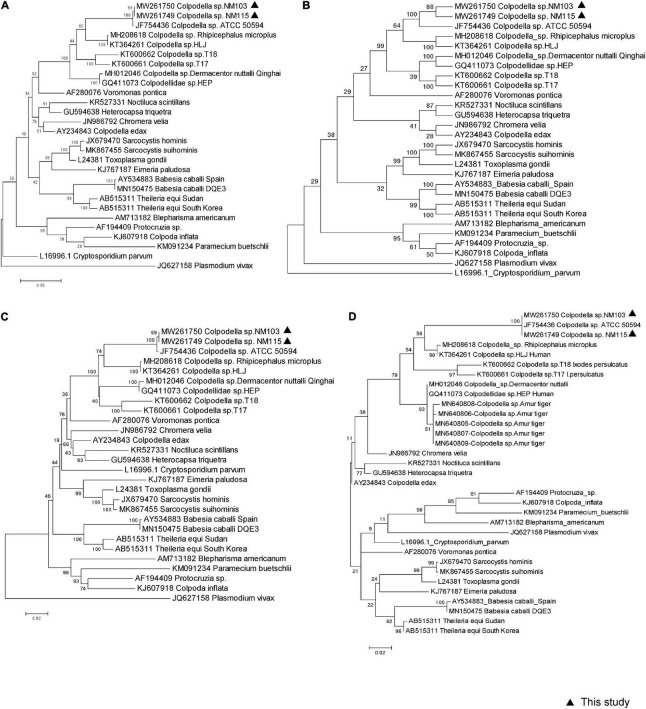
Molecular phylogenetic analysis of piroplasma from horse based on 18S rRNA sequences. Three methods were used to construct a phylogenetic tree, which are **(A)** Maximum-Likelihood Tree, **(B)** Maximum Parsimony Tree, **(C)** Neighbor Joining Tree based on near entire 18S rRNA sequences, respectively. **(D)** Maximum-Likelihood Tree based on 583 bp partial 18S rRNA sequences. All reference sequences show GenBank number and pathogen name, in which the sequence of this study is marked with a black triangle.

Two approximately 1,600-bp sequences (accession nos. MW261749 and MW261750) obtained from our sample NM103 and NM115 had three nucleotide bases difference, and were closely related to *Colpodella* sp. ATCC50594 strain (accession no. AY14207583) isolated from brown woodland soil at Gambrill State Park, located in Frederick, Maryland, with 99.18 and 98.73% similarity, respectively. We used the MegAlign component to perform multiple sequence alignments with the ClustalW algorithm to create the consensus phylogenetic tree based on nearly the entire 18S rRNA gene, along with other representative sequences from the GenBank database, respectively inferred by maximum likelihood, maximum parsimony, and the neighbor-joining method, with bootstrap values determined by 1,000 replicates in MEGA6.0 (Figure 2). Furthermore, the topology of all three trees were similar across methods. These two novel sequences fell within the same clade as *Colpodella* sp. ATCC50594, and represent the first evidence of *Colpodella* sp. detected in horses in China (Figures 2A–C). In addition, the consensus phylogenetic tree based on partial 18S rRNA gene, especially along with other representative sequences originated from Amur tiger deposited in GenBank database, inferred by maximum likelihood still confirmed the same topology and relationship (Figure 2D).

*Colpodella* spp. are uncommon in the natural environment according to the current research report; however, our present study detected two potential pathogenic agents, which were closely related to *Colpodella* sp. ATCC50594 isolated from soil and has possible links to the origin of intracellular parasitism (Getty et al., 2021), and close to *Colpodella* sp. HLJ (KT364261) and *Colpodella* sp. HEP (GQ411073) isolated from patients in China (Yuan et al., 2012; Jiang et al., 2018). Two *Colpodella* spp. were also found in positive *Ixodes persulcatus* ticks collected around the living area of the patients mentioned previously (Jiang et al., 2018). One of the *Rhipicephalus microplus* infesting cattle was also positive for *Colpodella* spp. (Matsimbe et al., 2017). In addition, the *Colpodella* sp. sequence under the accession number MH012044-46 was detected in *Dermacentor nuttalli*, which is commonly found in Inner Mongolia. Thus, it is likely that ticks might be important carriers of these microorganisms and serve as potential transmission vectors in both humans and animals. However, it remains unclear whether the ticks are mechanical or biological transmitters, which requires further study. On the basis of molecular biology, recent modified light microscopy using a new trichrome staining technique by Sam-Yellowe et al. (2020) will aid in diagnosis of infections of *Colpodella* sp. and provide another available avenue for future surveillance and research.

The two previous reports of human cases of *Colpodella* sp. infection presented with clinical symptoms similar to other erythrocyte parasitic infections, such as fever and hemolytic anemia (Yuan et al., 2012; Jiang et al., 2018). The Heilongjiang case presented with moderate nuchal rigidity neurological symptoms, with isolates obtained from the patient’s cerebral spinal fluid and from ticks collected in close proximity to the patient’s house.

In addition, five partial sequences of *Colpodella* sp. obtained from Amur tiger (*Panthera tigris altaica*) without other available detail information were also reported. The present study first showed that another vertebrate, an animal that has high contact with human and is often attached by ticks, is affected by *Colpodella* sp. with no obvious symptoms like human cases. However, it is certain that horses are an important risk factor or act as a reservoir for potential zoonotic pathogen *Colpodella* sp. infection in human. Extending investigation on them among vertebrates, vector ticks, and human is further needed.

## Conclusion

In conclusion, this study represents the first documented report of *Colpodella* sp. infection in horses. While the initial goal of our study was to investigate piroplasmosis in horses, our team inadvertently detected *Colpodella* sp. once 18S rRNA amplicons were subject to sequencing. In light of this, we believe *Colpodella* sp. should be considered a novel tick-borne zoonotic pathogen that has the potential to be mischaracterized as a piroplasmosis when screened with standard primers in the absence of sequence confirmation. However, it remains unknown what role horses play in disease maintenance, nor do we have a clear understanding of clinical symptoms associated with infection in horses, or if isolates detected in this study pose a risk to human health, warranting further investigation.

## Data Availability Statement

The datasets presented in this study can be found in online repositories. The names of the repository/repositories and accession number(s) can be found in the article/supplementary material.

## Author Contributions

JJ and JW designed the project and experiments. MX, YH, and HQ conducted the experiments. YH and MX analyzed the data. MX, YH, and JJ drafted the article. All authors contributed to the article and approved the submitted version.

## Conflict of Interest

The authors declare that the research was conducted in the absence of any commercial or financial relationships that could be construed as a potential conflict of interest.

## Publisher’s Note

All claims expressed in this article are solely those of the authors and do not necessarily represent those of their affiliated organizations, or those of the publisher, the editors and the reviewers. Any product that may be evaluated in this article, or claim that may be made by its manufacturer, is not guaranteed or endorsed by the publisher.
